# Identification of QTL Combinations that Cause Spikelet Sterility in Rice Derived from Interspecific Crosses

**DOI:** 10.1186/s12284-021-00540-6

**Published:** 2021-12-07

**Authors:** Chang-Min Lee, Jung-Pil Suh, Hyun-Su Park, Man-Kee Baek, O-Young Jeong, Song-Joong Yun, Young-Chan Cho, Suk-Man Kim

**Affiliations:** 1 Crop Breeding Division, National Institute of Crop Science, Rural Development Administration, Wanju, Republic of Korea; 2 Department of Crop Science and Biotechnology, Jeonbuk National University, Jeonju, Republic of Korea; 3 Department of Ecological and Environmental System, Kyungpook National University, Sangju, Republic of Korea

**Keywords:** Spikelet fertility, Spikelet sterility, QTL, Interspecies cross, Hybrid breakdown, Rice

## Abstract

**Background:**

The exploitation of useful genes through interspecific and intersubspecific crosses has been an important strategy for the genetic improvement of rice. Postzygotic reproductive isolation routinely occurs to hinder the growth of pollen or embryo sacs during the reproductive development of the wide crosses.

**Result:**

In this study, we investigated the genetic relationship between the hybrid breakdown of the population and transferred resistance genes derived from wide crosses using a near-isogenic population composed of 225 lines. Five loci (*qSS12*, *qSS8*, *qSS11*, *ePS6*-1, and *ePS6*-2) associated with spikelet fertility (SF) were identified by QTL and epistatic analysis, and two out of five epistasis interactions were found between the three QTLs (*qSS12*, *qSS8* and *qSS11*) and background marker loci (*ePS6*-1 and *ePS6*-2) on chromosome 6. The results of the QTL combinations suggested a genetic model that explains most of the interactions between spikelet fertility and the detected loci with positive or negative effects. Moreover, the major-effect QTLs, *qSS12* and *qSS8*, which exhibited additive gene effects, were narrowed down to 82- and 200-kb regions on chromosomes 12 and 8, respectively. Of the 13 ORFs present in the target regions, Os12g0589400 and Os12g0589898 for *qSS12* and OS8g0298700 for *qSS8* induced significantly different expression levels of the candidate genes in rice at the young panicle stage.

**Conclusion:**

The results will be useful for obtaining a further understanding of the mechanism causing the hybrid breakdown of a wide cross and will provide new information for developing rice cultivars with wide compatibility.

**Supplementary Information:**

The online version contains supplementary material available at 10.1186/s12284-021-00540-6.

## Background

Interspecific crosses have been continuously performed as effective counterplans to overcome the limited genetic diversity of cultivated rice (Tanksley and McCouch 1997). The introgression of useful genes or the emergence of new biotypes via recombination from various sources positively affect adaptation to the environment and sustain the potential for sustained genetic improvement over the long term. However, hybrid sterility, a postzygotic reproductive barrier, is quite common in hybrid plants, which cannot produce fertile pollen or embryo sacs during reproductive development (Fang et al. 2019; Ouyang 2019). Thus, the enlargement of a wide compatibility ability using molecular technology could be considered breaking the reproductive barrier. Using this strategy in rice breeding will lead to the utilization of rice and extension of the rice cultivation area, which will prevent obstacles due to environmental or genetic factors.

Plant species, including rice, are isolated by various types of reproductive barriers according to their life cycle, which prevents individuals of genetically diverged groups from mating, surviving or producing fertile offspring (Widmer et al. 2009; Rieseberg and Blackman 2010; Ouyang 2019). Postzygotic reproductive isolation (RI) occurring after mating is known to drive speciation and maintain species identity, which blocks or reduces the gene flow among species (Kubo et al. 2016). A common form of postzygotic RI appears to be abnormal in offspring generations, as demonstrated by hybrid breakdown/weakness, hybrid sterility, and necrosis in the F_1_, F_2_, or backcross generations (Jiang et al. 2008; Yamamoto et al. 2010; Ichitani et al. 2012; Ouyang and Zhang 2013). The syndrome is referred to as hybrid incompatibility proposed by the theory known as the “Bateson-Dobzhansky-Muller model” (Xie et al. 2019). Among the subtypes of postzygotic RI, hybrid necrosis and hybrid sterility have been recognized in F_1_ hybrids, whereas hybrid breakdown occurs in the F_2_ or later generations through a sterile or weakness phenotype (Yamamoto et al. 2010). To date, molecular genetic studies have revealed more than 50 loci that cause the fertility of inter(sub)specific crosses in rice, and these loci include loci with major effects and quantitative trait loci (QTL) with minor effects (Ouyang and Zhang 2013; Fang et al. 2019).

*S5*, *S7*, and *HSA1* have been cloned and characterized with regard to hybrid female sterility, and *Sa*, *Sc*, and *qHMS7* have also been characterized at the molecular level regarding male sterility. Hybrid sterility has been explained by two major genetic models: a one-locus allelic interaction model and a two-locus model (duplicate gametophytic lethal model) (Ouyang and Zhang 2013; Xie et al. 2019). In addition, sterility is mainly caused by the effect of multiple genes, which results in a very low pollen count and very low spikelet fertility, and sterility depends on a function of male gamete fertility, female gamete fertility, and affinity between the uniting male and female gametes (Song et al. 2005; Kubo et al. 2008). Additionally, there has been a study that cross-incompatibility in F_1_ hybrid may be associated with interspecific cross or genes involved in the immune response (Bomblies and Weigel 2007; Kubo et al. 2008). Epistatic interactions are considered to contribute substantially to variations in spikelet fertility (SF) in hybrid progeny. Hence, a better understanding of the genetic mechanism responsible for the rice incompatibility should also be identified and characterized precisely despite these complexities or low heritability.

In this study, we performed a QTL analysis to establish the relationship between SF and resistance genes (*R*-genes) in the near-isogenic line (NIL) population. Using the NIL population with allelic fragments obtained by inter(sub)specific crossing, we identified five loci (*qSS12*, *qSS8*, *qSS11*, *ePS6*-1, and *ePS6*-2) associated with SF and suggested a genetic model that explains the interactions within factors with positive or negative effects. The results showed that one combination (*SS12* + *SS8*) described 50% of the observed SF, and other interactions, including epistatic factors (*ePS6*-1 and *ePS6*-2), were responsible for the remaining SF. The analysis revealed the genetic basis of SF and will contribute to the development of intermediate parents or cultivars with a wide compatibility line using the favorite genes of distantly related species in our *japonica* rice breeding programs.

## Results

### Evaluation of SF Using the NIL Population

#### Development of the Population

Gene-pyramided line (GPL; a backcrossed line with the genetic background of Jinbu) is a NIL with the *japonica* background obtained from a marker-assisted backcross (MAB) based on an inter(sub)specific cross. Despite its advantage of multiple resistance, GPL is not optimal for practical use because it exhibits approximately 75% reduced fertility. Thus, we performed backcrossing again with Jinbu (*japonica*) to improve the SF (Additional file 1: Figure S1). Through foreground selection for each resistance gene (*R*-gene), ten cross plants were selected and advanced to generate an F_2_ population. From 240 F_2_ individuals, 225 lines were subsequently developed by measuring the main agronomic traits and performing marker selection for the *R*-genes and then advanced by single-seed descent (SSD).

#### Segregation Ratio of the SF

**Fig. 1:**
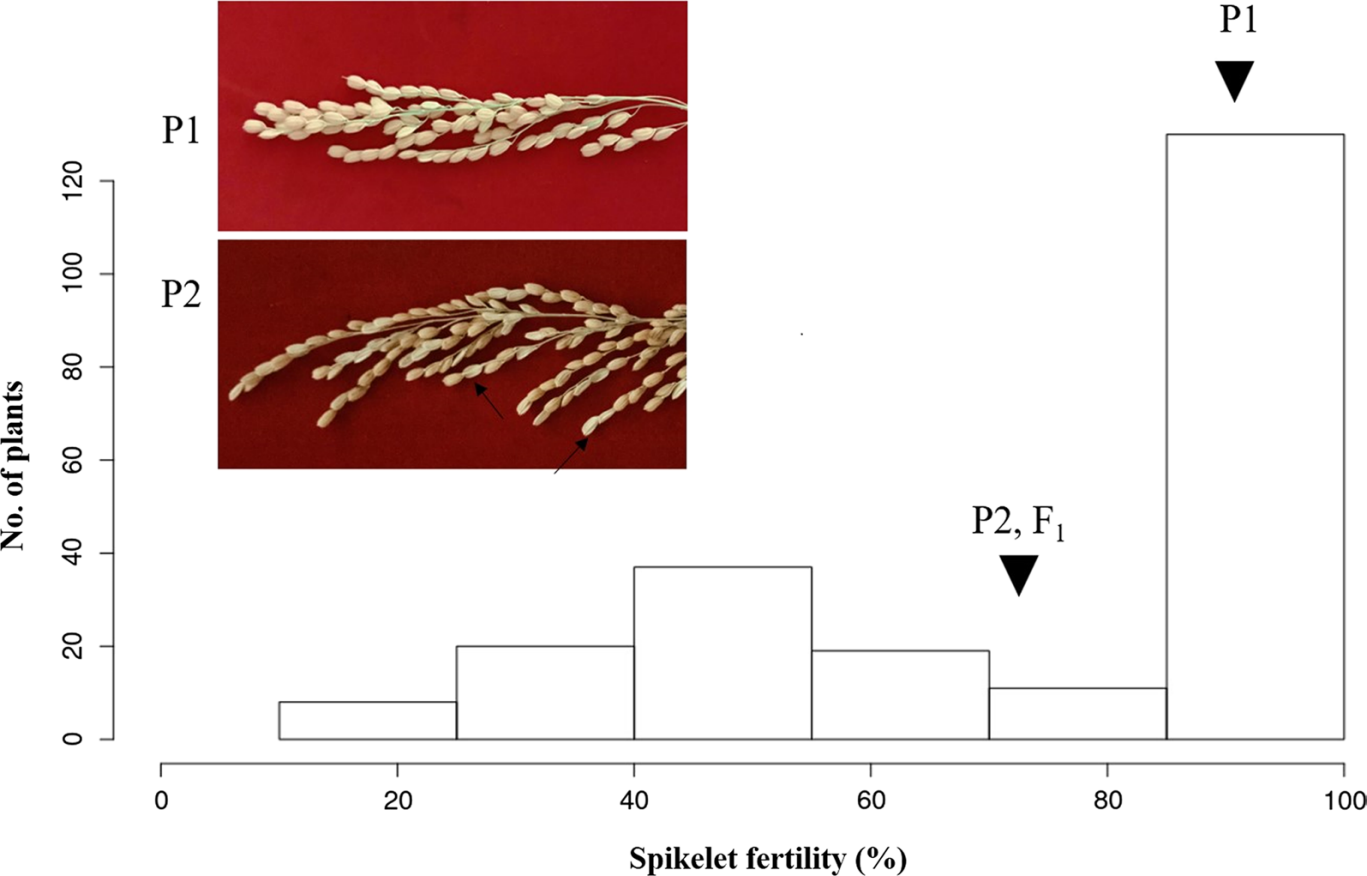
Frequency distribution of spikelet fertility among the 225 tested lines. F_1_ and the parent showed more than 75% spikelet fertility. P1 refers to Jinbu, and P2 is GPL

The SF of the developed 225 NILs was evaluated. The degree of SF ranged widely from 10 to 100% in the population and did not appear to exhibit a normal distribution curve on the tested trait (Fig. 1). While major agronomic traits showed a normal distribution pattern within the range of the parents at the same stage without symptoms of poor growth or inviability (data not shown). The segregation of the SF was analyzed to understand the inheritance pattern (Table 1). According to the reference point (RF), the segregation ratio was not fitting the radio of 1:2:1, but to fit 9:3:4 (F:M:S) at RF-I and RF-II (*X*^2^ for 9:3:4, *P* < 0.05). Because the SF of both parents was higher than 75%, the inheritance pattern of the NIL population suggested that multiple genes and not a single gene would dominate the SF.

**Table 1:** Segregation ratio of spikelet fertility (SS) in the tested 225 lines

Reference point (RF)	Seg	No. of plants				*X*^2^ value	
		Fertile	Moderate	Sterile	Total	1:2:1	9:3:4
RF-I	F:M:S	130	52	43	225	132.4**	5.49^ns^
RF-II	F:M:S	130	31	65	225	156.4**	4.97^ns^
RF-III	F:M:S	130	20	75	225	179.0**	18.01**

Seg.: segregation of spikelet fertile classified into three classes: F (Fertile), S (Sterile), and M (Moderate fertile) according to reference point (RP) distinguish fertility from sterility. RF-I (F: M: S = over 85%: 85–45%: less than 45%), RF-II (F: M: S = 85: 85–55: 55), RF-III (F: M: S = 85: 85–65: 65)

^ns^Not significant

Significant levels: *P < 0.05 and **P < 0.001

### Analysis of the Relationship Between the R-Genes and Spikelet Sterility (SS)

To identify the cause of occurrence of SS in the population, the relationship between the SF values and the introgression of *R*-genes was analyzed using correlation coefficients and ANOVA. The analysis revealed that *Bph18* only showed a weak positive correlation (*r* = 0.36) with SF, whereas other genes did not show any correlation with the trait (Additional file 1: Figure S2). In addition, an ANOVA F test for the three *R*-genes *Xa4*, *Pi40*, and *Bph18* was significant, providing no difference in interaction within the *R*-genes for SS in the population (Additional file 1: Table S1).

### Analysis of QTLs for SF

**Fig. 2:**
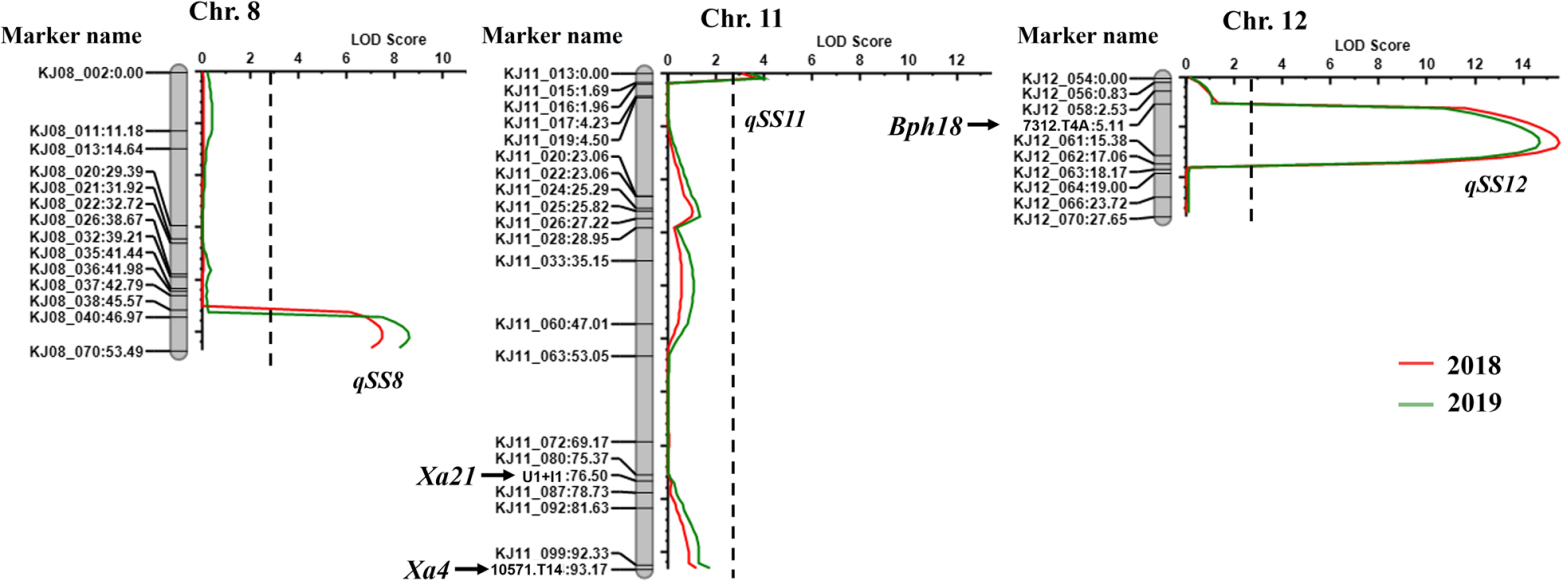
Genetic map and analysis of QTLs for spikelet fertility on chromosomes 8, 11, and 12. Three QTLs, *qSS8*, *qSS11*, and *qSS12*, were identified from 225 lines during 2018–2019. Three *R*-genes, *Xa4*, *Xa21*, and *Bph18*, were located on chromosomes 11 and 12, and only *Bph18* was within the region of *qSS12* associated with spikelet sterility. The vertical dotted lines represent the threshold ratio (LOD = 2.70) calculated by the permutation test with 1000 repetitions

QTL analysis was performed using a linkage map constructed using Kompetitive allele-specific PCR (KASP) marker sets (Additional file 2: Table S2). A total of 127 markers were eventually selected to be anchored on the 12 rice chromosomes (Additional file 1: Table S3). The linkage map showed an average distance of 8.62 cM within the flanking markers. The QTL analysis identified a total of three QTLs associated with SS through inclusive composite interval mapping (ICIM) and an empirical threshold of LOD > 2.70 (Table 2). The QTLs *qSS8*, *qSS11*, and *qSS12* on chromosomes 8, 11, and 12 were continuously detected for the experimental periods within the years 2018–2019 (Fig. 2). The QTLs revealed a negative influence on the fertility of the population. Of the two QTLs (*qSS12* and *qSS11*) derived from GPL, *qSS12*, with LOD scores of 15.5 and 14.7 within 7312.T4A (*Bph18*) and Kj12_061, was identified as the main QTL explaining 24.0% and 23.5% of the phenotypic variation (*R*^*2*^) in the ICIM analysis, respectively. Additionally, *qSS8* and *qSS11*, with LODs of 7.5–8.6 and 3.7–4.0, respectively, were detected within the flanking markers KJ08_040–JH08_070 and KJ11_013–KJ11-015 on chromosomes 8 and 11 with *R*^*2*^ values of 10.5–12.6 and 4.7–5.2%, respectively. The allele of *qSS8* was derived from Jinbu, and *qSS11*, similar to *qSS12*, was obtained from GPL.

**Table 2:** QTLs associated with spikelet fertility of 225 lines detected by composite and interval mapping

QTLs	Chr	2018				2019			
		Franking-markers	LOD	PVE (%)	Add	Franking-markers	LOD	PVE (%)	Add
*qSS8*	8	KJ08_40–KJ08_70	7.5	10.5	− 9.6	KJ08_40–KJ08_70	8.6	12.6	− 10.9
*qSS11*	11	KJ11_13–KJ11_15	3.7	4.7	7.0	KJ11_13–KJ11_15	4.0	5.2	7.8
*qSS12*	12	7312.T4A–KJ11_61	15.5	24.0	15.4	7312.T4A–KJ11_61	14.7	23.5	15.8

PVE (%): Percentage of phenotypic variation explained by the QTL, Add: additive effect

### Confirmation of the Effect of the QTL Combinations on SS

To verify the influence of the three QTLs for SS on the population, the combination effects were assessed by comparing the difference in the values to the number of QTLs detected in this study (Additional file 1: Figure S3). The results showed that the existence of QTL(s) divided the mean values of the SF of the tested QTL combinations into fertility and sterility. However, we also investigated the segregation pattern of the SF values in two cases (*qSS12* and *qSS8* + *qSS11*). The QTL combination composed of eight allelic types was explained by the allele type of the QTLs (Table 3). All the genotypes involved in TYPE_A belonging to the homozygote *qSS12*^*_*Jinbu^ exhibited high SF, regardless of the presence of the others. In TYPE_B without *qSS12*^_Jinbu^, all lines, including *qSF8*^*_*Jinbu^ in genotypes I (Type B-I) and II (Type B-II), showed high SS, whereas lines of genotypes III (Type B-III) and VI (TYPE B-VI) presented segregation patterns for SF ranging from 10 to 90%. The results explained that all the lines, including *qSS12*^*_*Jinbu^, showed normal fertility, whereas all the lines with the QTL combination (*qSS12*^*_*GPL^ + *qSS8*^*_*Jinbu^) continuously presented high SS in this population. In addition, given the segregation of SF observed, it could be assumed that other factors that had not yet been identified were involved epistatically, resulting in the occurrence of SS in the population.

**Table 3:** Spikelet fertility of the tested lines using the combinations of the three QTLs based on allele types detected from the parents

Genotypes	Allele type of *qSS12*	
	TYPE A (*qSS12*^_Jinbu^)	TYPE B (*qSS12*^_GPL^)
*qSS8*^_Jinbu^/*qSS11*^_GPL^	Fertile	Sterile
II. *qSS8*^_Jinbu^/*qSS11*^_Jinbu^	Fertile	Sterile
III*. qSS8*^_GPL^/*qSS11*^_Jinbu^	Fertile	Sterile and fertile
VI. *qSS8*^_GPL^/*qSS11*^_GPL^	Fertile	Sterile and fertile

### QTL-Background Loci Interaction for SS

An epistatic analysis was performed to identify the phenomenon of segregation in TYPE B_III and TYPE B_VI. Based on the analysis, digenic interactions were detected at five chromosomal positions with either negative or positive influences on SS (Additional file 1: Table S4). Two out of five epistasis interactions were found between the three QTLs (*qSS12*, *qSS8* and *qSS11*) and background marker loci on chromosome 6; the loci were renamed *ePS6*-1 and *ePS6-2*. The others were previously detected QTL-QTL interactions; a negative effect for SS was found within *qSS8*-*qSS12* and *qSS8*-*qSS11*, and a positive interaction was found in others. The phenotypic variation explained (PVE) by the epistatic QTLs ranged from 5.37 to 15.98% in the analysis. Moreover, *qSS8* on chromosome 8 exhibited digenic interactions with all loci with the exception of *ePS6*-1 on chromosome 6.

### Genetic Models for SF in the Lines

**Fig. 3:**
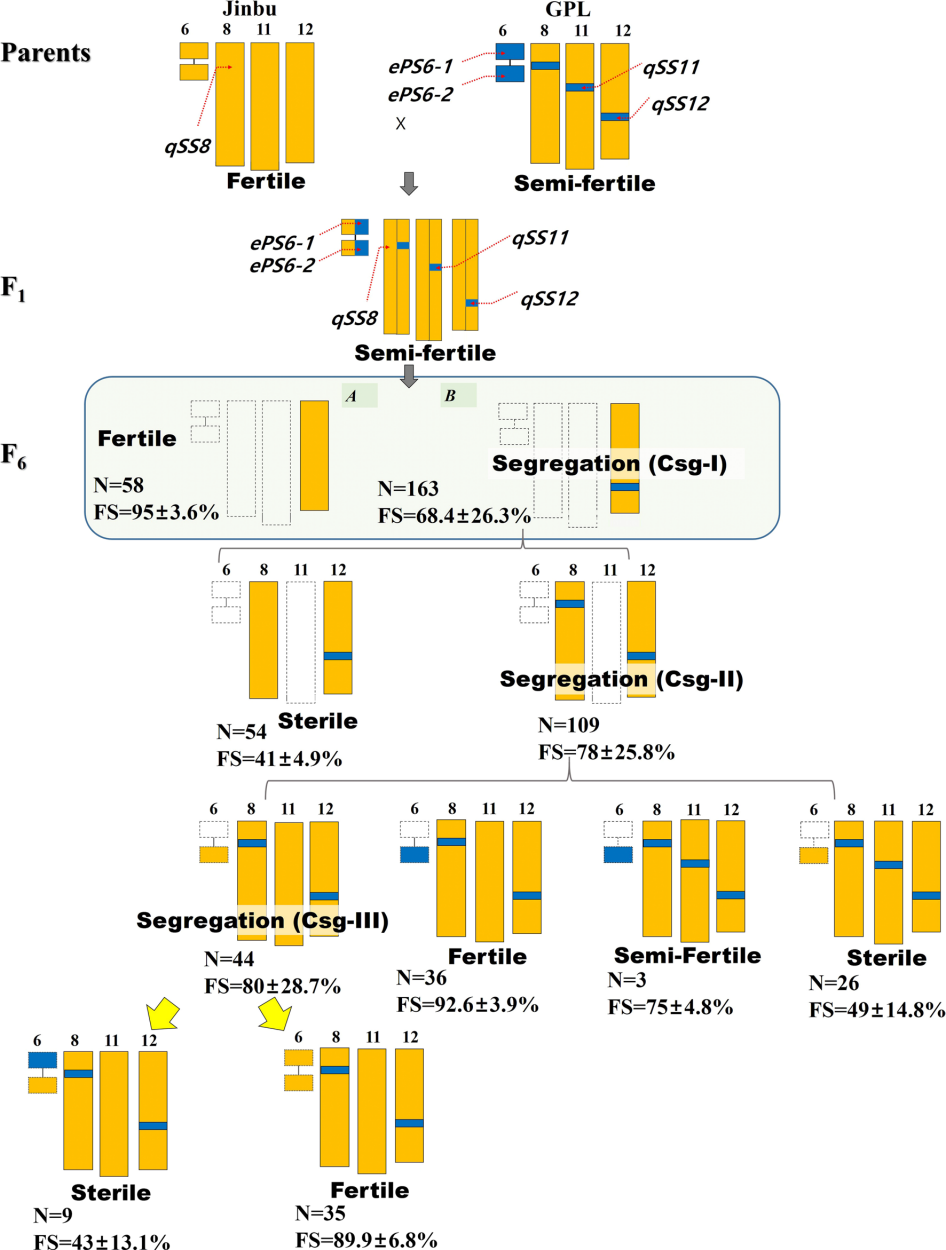
Genetic model for spikelet fertility explained by QTL combinations. The parent and F_1_ plants did not show phenomena related to spikelet sterility. The near-isogenic population showing the segregation of fertility in the F_2_ generation advanced six more generations. The orange plot indicates the allele of P1 (Jinbu), and the blue plot indicates P2 (GPL). The white rectangle of the dotted line indicates that it includes both alleles from the parents. Three cases (Csg-I, Csg-II, and Csg-III) for the segregation of SF were observed according to the combinations. N is number of plants, and FS is the fertility percentage

Using the results of the QTL and epistasis analyses, we constructed a genetic model to explain SS (Fig. 3). The procedure was performed by adding high-effect QTLs one by one in order. If a particular QTL combination exhibits a pattern of separating SF, it was added to the analysis, whether it matches the phenotype was assessed. Segregation issues were observed three times in the analysis, and the combinations were classified into Csg-I, Csg-II, and Csg-III in order. The combinations Csg-II (*qSS12*^*_*GPL^ + *qSS8*^_**GPL**^) were divided into four genotypes based on additional loci, *qSS11* and *ePS6-2*. In the combinations obtained by adding *qSS11*^_**GPL**^ to Csg-II, the SF phenotype was semi-fertile in the presence of *ePS6-2*^_GPL^ and sterile in the presence of *ePS6-2*^_Jinbu^, but in the case of Csg-II + *qSS11*^_**Jinbu**^, the phenotype was fertile in the presence of *ePS6-2*^_GPL^. Moreover, the combination Csg-III (Csg-II + *qSS11*^_Jinbu^ + *ePS6-2*^_Jinbu^) only showed a segregated pattern for SF. In this case, *ePS6-1* acted additively on *qSS12* to serve as a key factor in determining whether the phenotype was fertile or sterile. With the addition of *ePS6-1*^_GPL^ to Csg-III, the SF phenotype was sterile, and the SF in the absence of *ePS6-1*^_GPL^ was normal. The interaction of two QTLs, *qSS12* and *qSS8*, induced hybrid breakdown, which explained 50% of the observed SF in the population. Another 50% of the SF was explained by the interaction between other factors (*qSS11*, *ePS6-1*, and *ePS6-2*) and both QTLs.

### Physical Positioning of the Detected QTLs

**Fig. 4:**
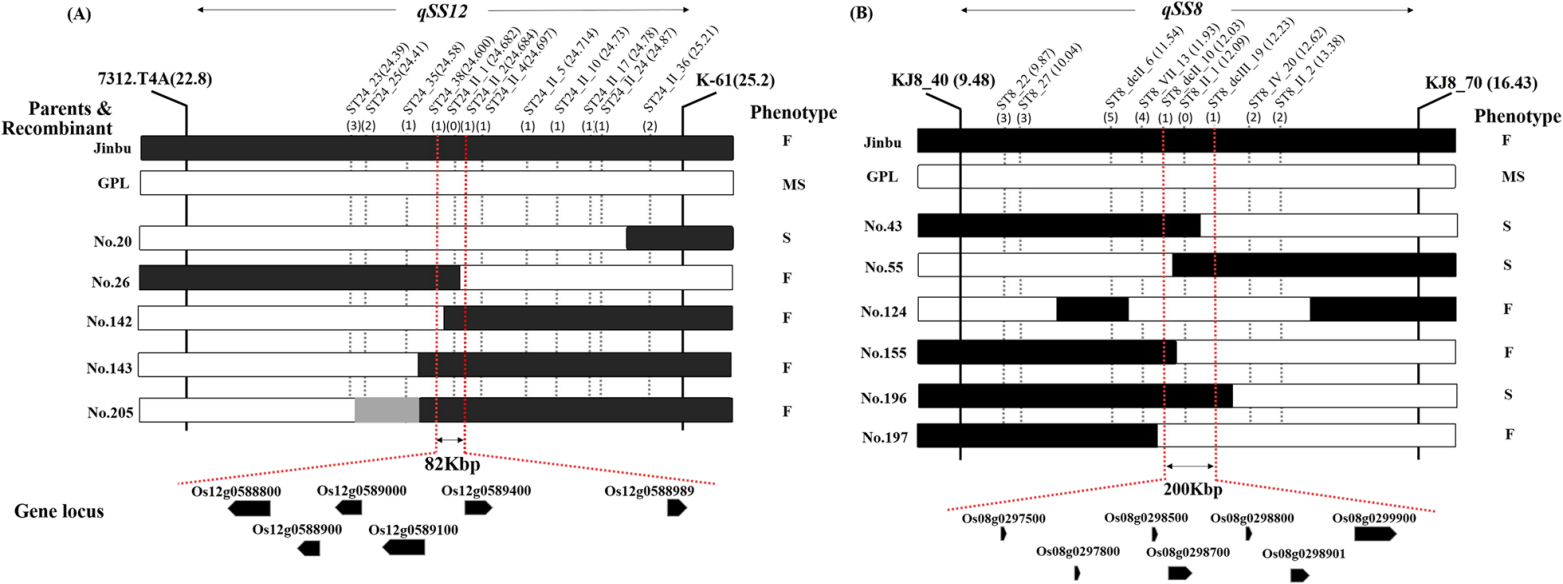
Fine mapping of the target regions of *qSS12* (**A**) and *qSS8* (**B**) on chromosomes 12 and 8, respectively. The black and white bars indicate homozygous alleles derived from Jinbu and GPL, respectively, and the gray bars show heterozygous alleles. The number in brackets indicates the number of recombinant events detected by discordance between genotype and phenotype

The target regions of *qSS12* and *qSS8* were narrowed down using each plant group selected as the recombinant and delimited within approximately 82-Kbp and 200-Kbp segments flanked by cleaved amplified polymorphic sequences (CAPS) and derived cleaved amplified polymorphic sequences (dCAPS) marker sets (Fig. 4). The recombinant lines showing discordance between phenotype and genotype were used to dissect the flanking region of the main QTL *qSS12*, which was found to be anchored from 22.8 to 25.2 Mbp on chromosome 12. A total of five recombinant plants selected by the flanking markers 7312.T4A (*Bph18*) and KJ12_061 were used to narrow down the target region (Fig. 4A). For dissection of the target QTLs, additional markers were newly designed according to the data from the whole-genome resequencing (WGR) analysis of the parents (Additional file 2: Table S5). Following the same procedures, fine mapping for the QTL *qSS8* was performed using the six recombinants (Fig. 4B and Additional file 2: Table S6).

Based on the results, the target region was narrowed to approximately 82-Kbp segments delimited by ST24_38 and ST24_II_2. Six ORFs based on Os-Nippobare-Reference-IRGSP-1.0 were identified in the target region (Additional file 2: Table S7). The position of *qSS8* was also narrowed down to identify the association with SS. The target region was flanked from approximately 200-Kbp segments delimited by ST8_dcII_10 and ST8_dcIII_19, and 7 ORFs were identified in the target region (Additional file 2: Table S7). From the fine-mapping results, we placed six and 13 ORF candidates in target regions for *qSS12* and *qSS8*, respectively.

### Expression of Genes by qPCR

**Fig. 5:**
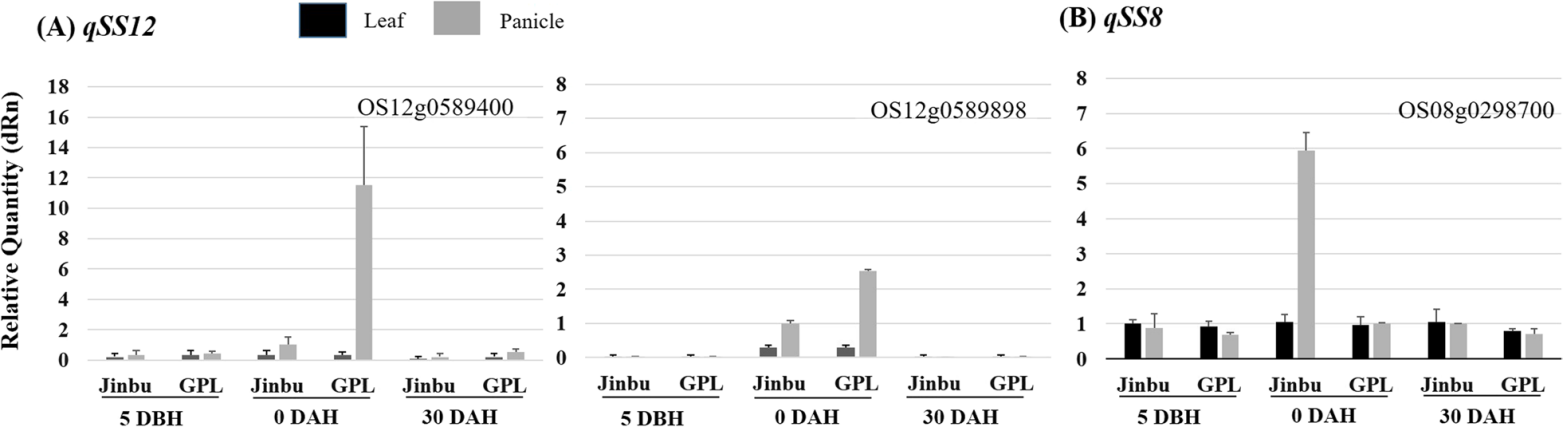
qPCR analysis of the gene expression pattern of Jinbu and GPL according to the panicle maturing stage. **A** Expression levels of two ORFs in the target region of *qSS12*. Panicles and leaves were collected from Jinbu and GPL at three time points: 5 days before heading (5 DBH), heading time (0 DAH), 30 days after heading (30 DAH). **B** Expression level of an ORF (OS08g0298700) in the target region of *qSS8* under conditions identical to those in A. The black and gray bars show the expression levels in leaves and panicles, respectively. The *eEF1-a* gene was used as an internal reference

We designed new primer sets to analyze the expression levels of 13 ORFs identified in the target regions on chromosomes 8 and 12 based on the Rice Annotation Project Database (RAP-DB, http://rapdb.dna.affrc.go.jp/). Of the six candidate genes for *qSS12*, two genes, Os12g0589400 and Os12g0589898, exhibited upregulated expression levels in only a panicle of GPL and were expressed at significantly higher levels in GPL than in Jinbu (Fig. 5A). An increase in the expression levels of the genes was observed in GPL at 0 days after heading (DAH), and the measured expression level was 11 times the concentration at 5 days before heading (DBH) and almost returned to its original level at 30 DAH. In the case of *qSS8*, however, the expression level of Os08g0298700 was upregulated in Jinbu at 0 DAH (Fig. 5B) to a sixfold higher concentration compared with that at 5 DBH and 30 DAH. The remaining ORFs revealed no significant difference between the parents. In particular, the qPCR analysis using lines with both QTLs *qSS12* and *qSS8* showed that both genes were expressed independently with no interaction at the RNA level (data not shown).

## Discussion

The inter(sub)specific cross is often considered for exploiting strong heterosis or overcoming the limitations of the genetic background, particularly in *japonica* breeding programs against various stresses (Ouyang 2019). However, the reduced fertility or weakness observed in the cross serves as a barrier to the further utilization of a wide range of subjected advantages. In this study, we performed a QTL analysis to identify the cause of SS observed in 225 NILs derivatized from a cross between the *japonica* cultivar Jinbu and the introgression line (IL) GPL with a *japonica* genetic background (Additional file 1: Figure S1). In a preliminary study, F_1_ plants derived from a cross between Jinbu and GPL were obtained to improve the SF of the IL as part of a breeding program, and the resulting plants exhibited approximately 75% SF under field conditions. At the F_1_ stage, no poor characteristic phenotype, called postzygotic isolation, was observed, whereas wide-ranging segregation manifested in the F_2_ generation through the sterility phenotype. This hybrid breakdown is one of the postzygotic isolations observed in F_2_ or later generations produced by interspecific crosses (Yamamoto et al. 2010). In this study, we transferred five *R*-genes into the *japonica* cultivar Jinbu to develop multiple resistant cultivars by interspecific crossing; however, we observed hybrid breakdown in the segregating generation. In fact, hybrid breakdown causing high sterility shows typical polygenic inheritance; hence, it is conceivable that the interaction among the polygenes is related to the sterility mechanism (Kubo et al. 2008).

To confirm whether the issue was caused by pyramiding *R*-genes, statistical analyses were performed using the NILs developed in this study (Additional file 1: Figure S1 and Table S1). The results revealed that SS was not fully interpreted by a single gene or combinations of genes, with the exception of *Bph18* on chromosome 12, which showed a weak positive correlation (*r* = 0.36) with the trait. *Bph18* conferring resistance to brown plant hopper (BPH) encodes the NBS-NBS-LRR protein in the endomembranes in a cell, and the gene might recognize BPH invasion at endomembranes in phloem cells (Ji et al. 2016). According to previous reports, NBS-LRR triggers the immune response signal in response to pathogen attack and is involved in an effector-triggered immunity system in which the NBS-LRR recognizes avirulence proteins either directly or indirectly (Chisholm et al. 2006; DeYoung and Innes 2006).

Through linkage analysis, we identified QTLs associated with SS, which was assumed to be the cause of the deleterious interaction with the transferred *R*-gene *Bph18.* In the analysis, three QTLs, *qSS8*, *qSS11*, and *qSS12,* were identified on chromosomes 8, 11 and 12, respectively (Table 2 and Fig. 2). Based on the analysis, all QTLs associated with SS had LOD values of 25.8–26.6 and explained 35.9–38.4% of the phenotypic variation within two years by CIM. Further epistatic QTL analysis allowed us to identify two additional loci (*ePS6-1*^_GPL^ and *ePS6-2*^_GPL^) on chromosome 6. To date, hybrid sterility or weakness has mainly been thought to be caused by one-locus allelic interactions at a single locus and a two-locus model (Chen et al. 2008; Long et al. 2008; Kubo et al. 2016; Shen et al. 2017; Xie et al. 2017; Koide et al. 2018). In addition, the accumulated effect of multiple loci has been widely reported to be associated with weakness or SF in rice (Wang et al. 1998; Yamamoto et al. 2007; Kubo et al. 2011). In fact, the interaction among polygenes is related to the sterility mechanism and exhibits typical polygenic inheritance (Zhao et al. 2007; Kubo et al. 2008). Song et al. (2005) identified six QTLs on chromosomes 5, 6, 8, and 12 that control the SF of intersubspecific hybrids of rice. Kubo and Yoshimura (2005) reported three loci designated *hsa1*, *hsa2*, and *hsa3* on chromosomes 12, 8 and 9 that are involved in hybrid breakdown by female gametes in a *japonica*-*indica* cross.

Based on the analysis of QTL combinations, we established a genetic model that explains the interaction among five loci (*qSS12*^_GPL^, *qSS8*^_Jinbu^, *qSS11*^_GPL^, *ePS6-1*, and *ePS6-2*) associated with SF. The combination composed of the two loci *SS12* (*qSS12*^_GPL^) and *SS8* (*qSS8*^_Jinbu^) exerted the most dominant effects on SF in the analysis. *qSS12*^*_*GPL^, which is named *SS12* on chromosome 12, is the main QTL associated with SF. Considering the fertility level of the parent GPL, *qSS8*^_GPL^ appears to inhibit or hinder the role of *SS*12, or *qSS8*_^Jinbu^ appears to strengthen the role in triggering SF. The reason for this finding is that all the plants in which *qSS8*^_GPL^ was replaced by *qSS8*^_Jinbu^, including the combination (*qSS12*^*_*GPL^ + *qSS8*^_Jinbu^), showed severe sterility (Table 3). Despite their high involvement in sterility, the combination explained only approximately 50% of the SF in the population. The remaining SF could be explained by *ePS6-1* and *6-2* on chromosome 6 with more complex interactions. *ePS6-2* interacted with *qSS8*^_GPL^ and was negatively affected by SS, whereas *ePS6-1* interacted with *qSS12*^_GPL^ and was positively involved in SS. The case of *qSS11*^_GPL^ also showed an association with sterility, which indicated an additive effect on the tested trait. Based on the results, we could clearly explain most of the SF in the population using the five loci detected in this study. Moreover, the main QTL combination (*SS12* + *SS8*) in this study tended to be similar to epistasis by *hsa1* and *has2* (Kubo and Yoshimura 2005).

We performed fine mapping to each region of the main QTLs (*qSS12* and *qSS8*) for SS in the population using WGR data. From the results we could delimited the targets within 82-Kbp and 200-Kbp segments on each chromosome. Contrary to our expectations, however, no known *R*-genes were identified. In conclusion, *Bph18* was not included in the range of *qSS12*, and then the SS was not related to the autoimmune response by the *R*-genes introgression. It is simply a result of being transferred to epistasis together, causing SS during hybridization because of their close physical location.

To confirm the candidate gene(s) for *SS12* and *SS8*, qPCR was conducted using primer sets developed based on the sequences in exons. For SS12, the expression levels of only two genes, Os12g0589400 and Os12g0589898, were significantly higher in GPL than in Jinbu, as determined by qPCR (Fig. 5A). Both ORFs encoded “Domain of unknown function (DUF1618 domain)-containing protein” and “Similar to 2-isopropylmalate synthase B” as putative functions, respectively. *SS12* was identified at the position of *hsa1* containing *HSA1a* and *b*, which is known as the hybrid sterility gene (Kubo et al. 2016). The genetic positions occupied by the two genes appear to be identical and performed functions negatively associated with the fertility of rice, even though both were derived from different allelic sources, i.e., *SS12* and *hsa1* originated from *O. australiensis* and *O. sativa indica*, respectively. Moreover, among the candidates for *SS8*, the expression level of only OS08g0298700 was upregulated in the panicle of Jinbu at 0 DAH (Fig. 5B), and the ORF was known to encode “Similar to male sterility protein 2”. However, the actual reason for SS determined in this study was far from the problem with pollen (Additional file 1: Figure S4). *SS12* and *SS8* were independently expressed in the plants with each corresponding allele, regardless of whether the plant’s phenotype was fertile or sterile. This fact suggested that the two genes did not appear to affect each other’s expression. Given the discrepancy between gene expression and phenotype, we hypothesize that SS could be caused by interactions within encoded proteins or by additive accumulation of each QTL effect.

## Conclusions

In this study, we identified novel gene combinations controlling SS in a NIL population with a *japonica* genetic background derived from an interspecies cross. Based on the results, the combinations composed of the five loci were involved in SS either positively or negatively through interactions among the loci. Additionally, a genetic model explaining a set of complementary genes controlling hybrid breakdown in rice was suggested by the results from the QTL and epistatic analyses. The current results facilitate the establishment of compatible cultivars using PCR-based markers developed in this study. Furthermore, a functional analysis of the identified genes will be performed in a follow-up study. A mechanistic understanding of incompatibilities, such as hybrid breakdown caused by a wide cross, will help increase the availability of various useful genetic sources for rice breeding programs, and the approach performed in this study will be more effective for *japonica* cultivars with a relatively narrow genetic background.

## Materials and Methods

### Plant Materials and Mapping Population

The *japonica* cultivar Jinbu and the GPL were used as the parental cultivars for the mapping population. Jinbu is an early-maturing cultivar with cold tolerance and good grain quality. The other parent, GPL, has five different *R*-genes introduced by inter(sub)specific crosses (Additional file 1: Figure S5) (Suh et al. 2015). Three *R-*genes, *Xa4*, *xa5*, and *Xa21*, for bacterial leaf blight resistance were introgressed from a donor derived from *O. sativa* subsp. *Indica*, and two *R-*genes, *Pi40* and *Bph18*, for blast and BHP, respectively, were introduced by two lines, IR65482-4-136-2-2 and IR65482-7-216-1-2, which are resistance donors derived from *O. australiensis*. To increase the SF of GPL, GPL was backcrossed again with Jinbu, and 225 NILs were developed using a single descent method. The population based on the *japonica* background was used to access the phenotypic data and to construct a molecular genetic map for identification of QTLs controlling SF.

### Evaluation of the SF Rate

The population was cultivated and evaluated in an experimental field at the National Institute of Crop Sciences (NICS), RDA, Wanju, South Korea, from 2018 to 2019. For evaluation of the fertility rate, three panicles with fully ripened grains were collected from the plants, and the numbers of filled and unfilled spikelet seeds were counted. The fertility rate was estimated using the following formula: fertility rate = number of filled grains/total number of filled and unfilled grains (Kubo et al. 2016). To distinguish fertility and sterility, we needed to identify a RF for determining a criterion for SF in the population because the value of SF was continuously obtained from the phenotypic evaluation. After a fixed fertility level of over 85%, the RF was divided into three classes based on moderate SF (MSF) levels: RF-I (S:M:F = less 45%:45–85%:over 85%), RF-II (F:M:S = less than 55%:55–85%:over 85%) and RF-III (F:M:S = less than 65%:65–85%:over 85%).

### Genotyping and Linkage Mapping

For QTL analysis, we used a total of 771 KASP markers developed to detect single nucleotide polymorphisms (SNPs) of Korean *japonica* rice varieties (Cheon et al. 2018). To confirm that the markers were anchored on each chromosome, some SNPs among the genotypic datasets were removed if biased segregation appeared within the SNPs at overlapping positions on the chromosomes. The selected KASP markers were used to construct a genetic linkage map using the linkage mapping software QTL IciMapping, version 4.1 (Meng et al. 2015).

### Data Analysis

To determine the relationship between SF and the *R*-genes introduced into the correlation analysis, the corrplot package in R was used. Two-way ANOVAs were conducted to study the main effects of the five resistance markers and their interactions using R version 4.0.2. QTL analysis and epistatic mapping were conducted using conventional mapping for inclusive composite interval mapping for additive QTLs (ICIM‐ADD) (Meng et al. 2015). Permutation tests with 1000 replicates (*P* ≤ 0.05) were applied to confirm the significant threshold values of the LOD scores for QTL detection (Churchill and Doerge 1994). The epistatic interactions between marker loci of RILs for SF were analyzed using the ICIM-EPI functions in QTL IciMapping version 4.1. A threshold LOD of 4.0 with probability values for entering variables (PIN) of 0.0001 was used to define the significant epistatic QTLs (Chattopadhyay et al. 2019).

### Development of DNA Markers and Whole-Genome Resequencing (WGR)

For fine mapping, we designed new primer sets to narrow down the target region using the WGR data of the parents. Particularly for CAPS and dCAPS, specific restriction sites in the target region were detected using the marker design software RICE SNP-MINER. WGR was performed using an Illumina NovaSeq 6000 system (Illumina, USA) following the provided protocols for 2 × 100 sequencing. The DNA library was prepared according to the TruSeq Nano DNA library preparation protocol (Cat. No. FC-121-4001).

### qPCR Analysis

The real-time PCR array was performed using an Exicycler™ 384 Real-Time Quantitative Thermal Block (Bioneer, Daejeon, Korea) with the following cycling parameters: 40 cycles of 95 °C for 5 s, 58 °C for 25 s and 72 °C for 30 s. Data analysis was performed based on the relative quantitative method, and the ΔΔCT value was used to determine the relative fold change in expression. All the data were normalized to the expression level of the reference gene Os03g08010 (*eEF1-a*).

## Supplementary Information

**Additional file 1**. **Figure S1**. Procedure for the development of NILs by single seed descent (SSD). Phenotypic and genotypic selections were performed to select promising advanced backcrossed lines, and the lines were subjected to a bioassay for bacterial blight, BPH and blasts. **Figure S2**. Correlation efficiency of five *R*-genes related to the spikelet fertility of the tested lines. **Table S1**. ANOVA of resistance genes and their interactions on spikelet fertility. **Table S3**. Substituted chromosome segments from donor parents in gene pyramided lines by KASP marker analysis. **Figure S3**. Notched box plots for spikelet fertility (SF) of lines grouped by each QTL-QTL combination. The different letters show significant differences in SF among the tested lines based on Duncan’s multiple range test. Means followed by the same letter are not significantly different at the 5% significance level. **Table S4** Epistatic interactions among major effect loci of spikelet sterility. **Figure S4**. Pollen grains obtained from the parents, sterility, and fertility lines at the flowering stage. Pollen grains were stained with 1% iodine-potassium iodide (I2-KI) on glass slides. Scale bars = 50μm. **Figure S5**. Graphical genotype of the parents, Jinbu (P1) and GPL (P2) using KASP marker set. The horizontal lines on each chromosome of P2 indicate sites of segments introgressed from R-donor lines. Genetic background of both was derived from Jinbu. The five R-genes were marked in the box on the chromosome of P2.

**Additional file 2**. **Table S2**. List of KASP and DNA markers used in this study. **Table S5**. Primer sequences for qSS12 used in this study. **Table S6**. Primer sequences for qSS8 used in this study. **Table S7**. The putative ORFs in the two target regions of SS12 and SS8 based on the annotation data on Os-Nipponbare-Reference-IRGSP-1.0.

## Data Availability

Not applicable.
