# Supplementary material for: Identification of QTL Combinations that Cause Spikelet Sterility in Rice Derived from Interspecific Crosses
Source: Rice (N Y). 2021 Dec 7;14:99. doi: 10.1186/s12284-021-00540-6 (PMC8651928; doi:10.1186/s12284-021-00540-6)
Supplement: Supplementary file 1 — Additional file 1. Figure S1. Procedure for the development of NILs by single seed descent (SSD). Phenotypic and genotypic selections were performed to select promising advanced backcrossed lines, and the lines were subjected to a bioassay for bacterial blight, BPH and blasts. Figure S2. Correlation efficiency of five R-genes related to the spikelet fertility of the tested lines. Table S1. ANOVA of resistance genes and their interactions on spikelet fertility. Table S3. Substituted chromosome segments from donor parents in gene pyramided lines by KASP marker analysis. Figure S3. Notched box plots for spikelet fertility (SF) of lines grouped by each QTL-QTL combination. The different letters show significant differences in SF among the tested lines based on Duncan’s multiple range test. Means followed by the same letter are not significantly different at the 5% significance level. Table S4 Epistatic interactions among major effect loci of spikelet sterility. Figure S4. Pollen grains obtained from the parents, sterility, and fertility lines at the flowering stage. Pollen grains were stained with 1% iodine-potassium iodide (I2-KI) on glass slides. Scale bars = 50μm. Figure S5. Graphical genotype of the parents, Jinbu (P1) and GPL (P2) using KASP marker set. The horizontal lines on each chromosome of P2 indicate sites of segments introgressed from R-donor lines. Genetic background of both was derived from Jinbu. The five R-genes were marked in the box on the chromosome of P2. [file 12284_2021_540_MOESM1_ESM.docx]

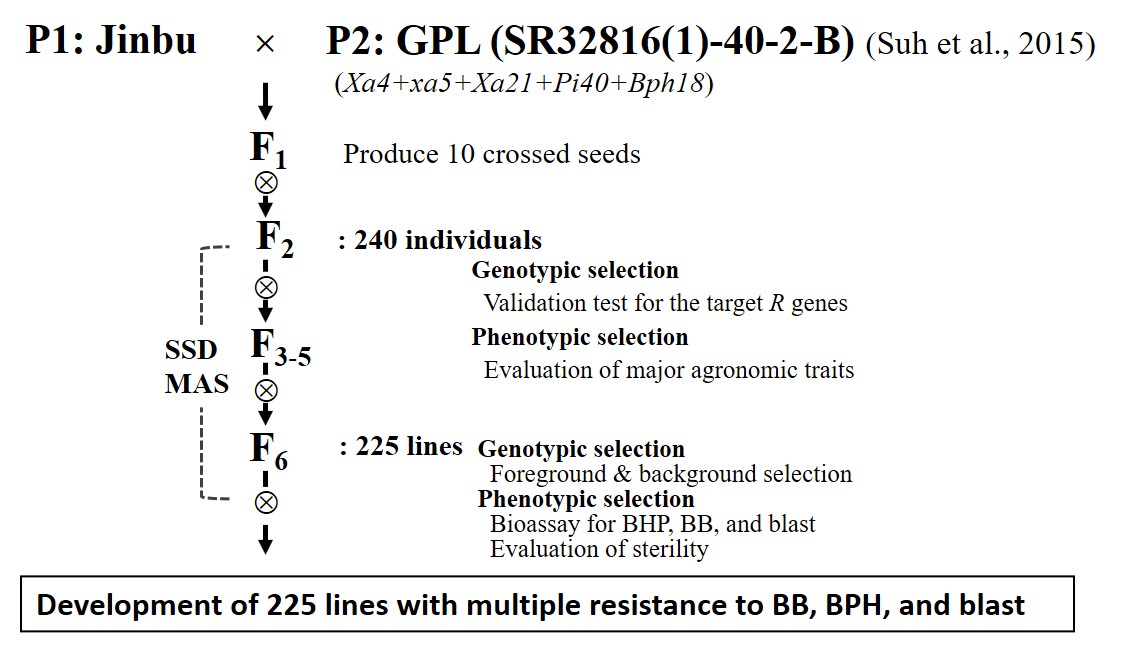


Additional file 1: Figure S1. 1 Procedure for the development of NILs by single seed descent (SSD). Phenotypic and genotypic selections were performed to select promising advanced backcrossed lines, and the lines were subjected to a bioassay for bacterial blight, BPH and blast.


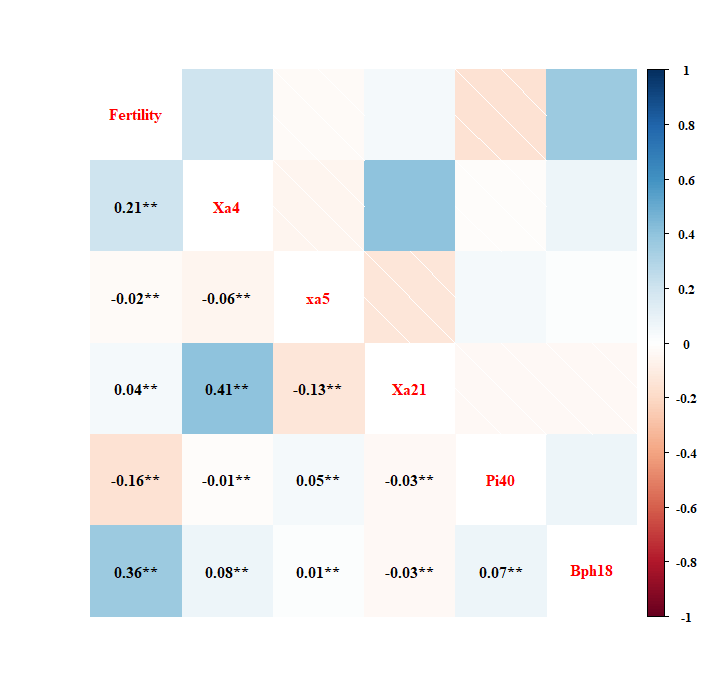


Additional file 1: Figure S2. Correlation efficiency of five *R*-genes related to the spikelet fertility of the tested lines.

| Genotype | Df | SS | MS | F value |
| --- | --- | --- | --- | --- |
| *Xa4* | 1 | 5418 | 5418 | 9.85** |
| *xa5* | 1 | 51 | 51 | 0.09^ns^ |
| *Xa21* | 1 | 1250 | 1250 | 2.27 ^ns^ |
| *Pi40* | 1 | 3390 | 3390 | 6.16* |
| *Bph18* | 1 | 17371 | 17371 | 31.57*** |
| *Xa4:xa5* | 1 | 182 | 182 | 0.33 ^ns^ |
| *Xa4:Xa21* | 1 | 939 | 939 | 1.71 ^ns^ |
| *xa5:Xa21* | 1 | 27 | 27 | 0.05 ^ns^ |
| *Xa4:Pi40* | 1 | 238 | 238 | 0.43 ^ns^ |
| *xa5:Pi40* | 1 | 1423 | 1423 | 2.59 ^ns^ |
| *Xa21:Pi40* | 1 | 51 | 51 | 0.09 ^ns^ |
| *Xa4:Bph18* | 1 | 1976 | 1976 | 3.59 ^ns^ |
| *xa5:Bph18* | 1 | 193 | 193 | 0.35 ^ns^ |
| *Xa21:Bph18* | 1 | 2101 | 2101 | 3.82 ^ns^ |
| *Pi40:Bph18* | 1 | 1702 | 1702 | 3.09 ^ns^ |

Additional file 1: Table S1 ANOVA of resistance genes and their interactions on spikelet fertility

^ns^: not significant

Significant levels: *^*^P*<0.05 and *^**^P*<0.001.

Additional file 1: Table S3. Substituted chromosome segments from donor parents in gene pyramided lines by KASP marker analysis.

| Chr.^a^ | No. of markers | No. of polymorphic markers^b^ | | No. of markers selected by QTL analysis | Interval (cM)^d^ | Chr. length (cM)^e^ |
| --- | --- | --- | --- | --- | --- | --- |
| 1 | 102 | 30 | | 14 | 8.57 | 111.45 |
| 2 | 66 | 0 | | - | - | - |
| 3 | 48 | 8 | | 7 | 20.55 | 123.33 |
| 4 | 60 | 13 | | 9 | 9.25 | 73.99 |
| 5 | 63 | 23 | | 12 | 8.91 | 98.09 |
| 6 | 61 | 15 | | 12 | 12.26 | 134.89 |
| 7 | 68 | 15 | | 9 | 9.40 | 75.17 |
| 8 | 67 | 16 | | 14 | 4.11 | 53.49 |
| 9 | 64 | 20 | | 12 | 3.15 | 34.66 |
| 10 | 44 | 16 | | 7 | 10.97 | 65.82 |
| 11 | 74 | 30 | | 21 | 4.66 | 93.17 |
| 12 | 54 | 10 | | 10 | 3.07 | 27.65 |
| Total (Av.) | 771 | 196 | | 127 | (8.62) | 891.71 |
| ^a^ Chromosome number | | | | | | |
| ^b^ Number of markers showing polymorphic patterns between Jinbu and GPL | | | | | | |
| ^c^ Percentage of polymorphism between Jinbu and GPL | | | | | | |
| ^d^ Average marker interval (cM) | | |  |  |  |  |
| ^e^ Chromosome length in centimorgans (cM) | | | |  |  |  |


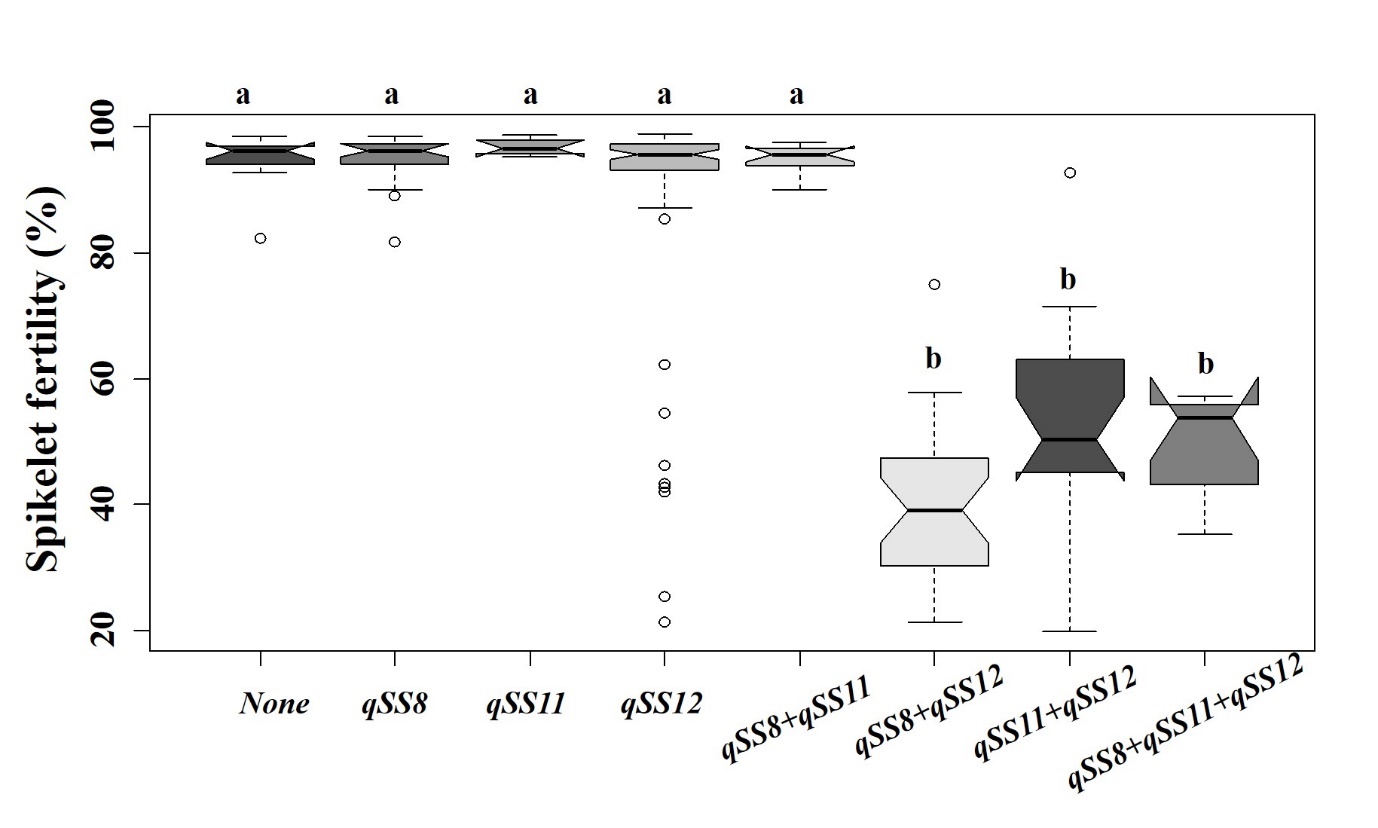


Additional file 1: Figure S3. Notched box plots for spikelet fertility (SF) of lines grouped by each QTL-QTL combination. The different letters show significant differences in SF among the tested lines based on Duncan’s multiple range test. Means followed by the same letter are not significantly different at the 5% significance level.

Additional file 1: Table S4. Epistatic interactions among major effect loci of spikelet sterility

| Locus 1 | | | |  | Locus 2 | | | | LOD | PVE  (%) | Add^b^ | Add^c^ | Add by Add |
| --- | --- | --- | --- | --- | --- | --- | --- | --- | --- | --- | --- | --- | --- |
| Chr. ^a^ | Position | Left marker  (Position) | Right marker  (Position) |  | Chr. | Position | Left marker  (Position) | Right marker  (Position) |  |  |  |  |  |
| 6 | 98 | 9871.T7E2b | KJ06_045 |  | 8 | 48 | KJ08_040 | KJ08_070 | 4.05 | 5.37 | 1.51 | -12.84 | 8.56 |
| 6 | 24 | KJ06_008 | KJ06_017 |  | 11 | 0 | KJ11_013 | KJ11_015 | 5.85 | 7.88 | 5.48 | 3.06 | 12.65 |
| 8 | 48 | KJ08_040 | KJ08_070 |  | 11 | 4 | KJ11_015 | KJ11_017 | 8.53 | 6.35 | -5.42 | -0.26 | -11.09 |
| 6 | 20 | KJ06_008 | KJ06_017 |  | 12 | 12 | 7312.T4A | KJ12_061 | 7.95 | 15.98 | 10.83 | 19.58 | -9.51 |
| 8 | 50 | KJ08_040 | KJ08_070 |  | 12 | 12 | 7312.T4A | KJ12_061 | 8.98 | 15.51 | -11.45 | 18.07 | 10.96 |

^a^ Chromosome

^b^ Estimated additive effect of position 1

^c^ Estimated additive effect of position 2


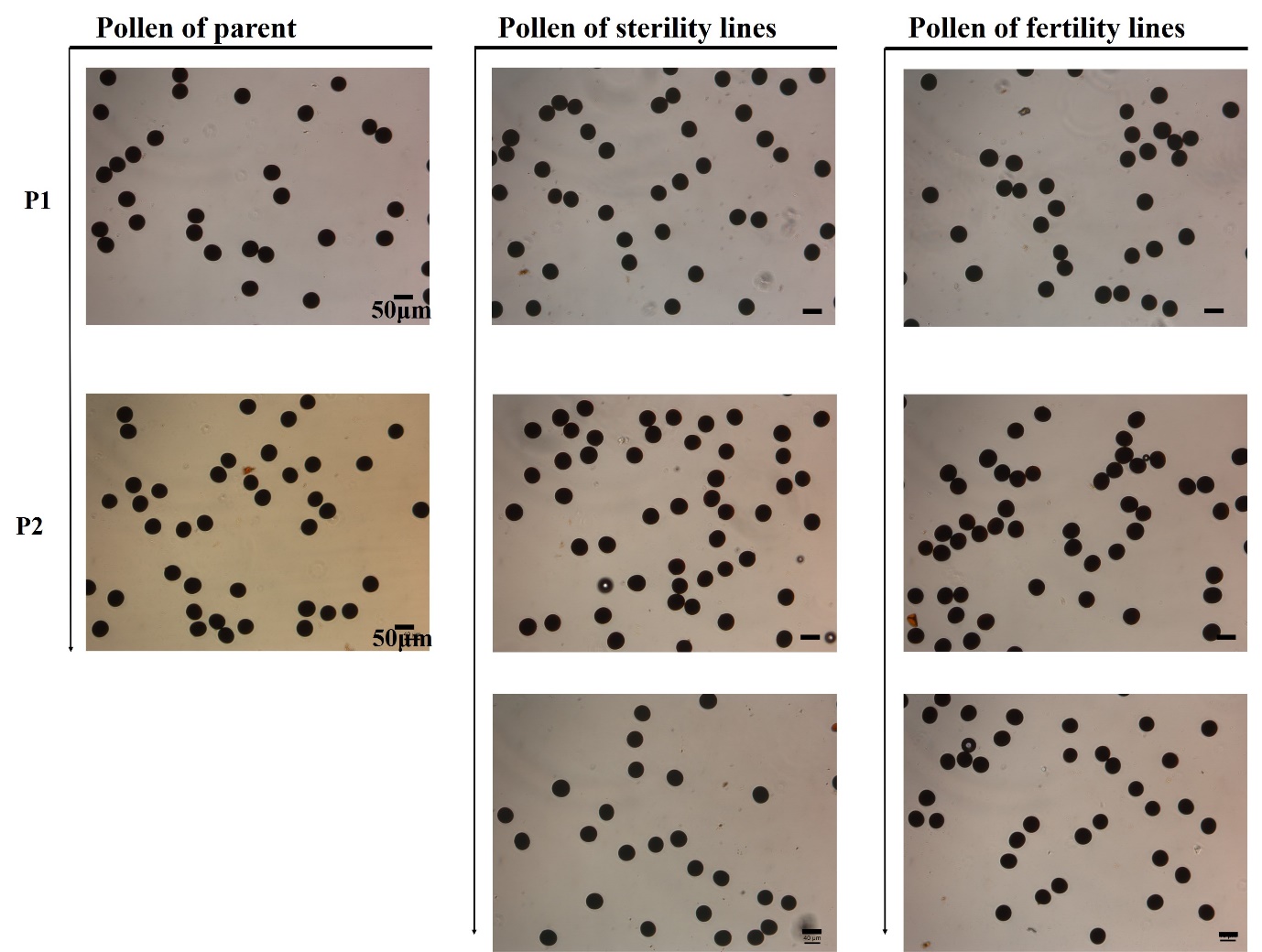


Additional file 1: Figure S4. Pollen grains obtained from sterility and fertility lines at the flowering stage. Each line is representing the pollens selected from different lines according to the SS phenotype. Pollen grains were stained with 1% iodine-potassium iodide (I_2_-KI) on glass slides. Scale bars = 50μm.


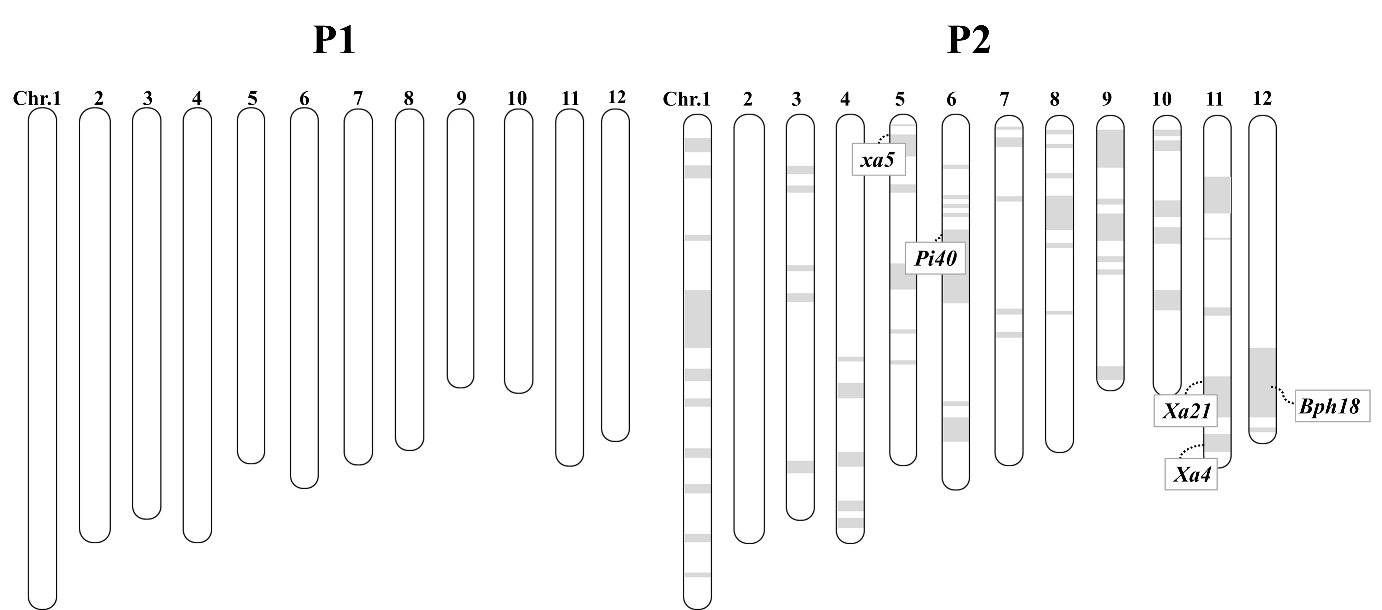


Additional file 1: Figure S5. Graphical genotype of the parents, Jinbu (P1) and GPL (P2). The horizontal lines on each chromosome of P2 indicate sites of segments introgressed from *R*-donor lines. Genetic background of both was derived from Jinbu. The five *R*-genes were marked in the box on the chromosome of P2.
